# Using Gaussian process for velocity reconstruction after coronary stenosis applicable in positron emission particle tracking: An *in-silico* study

**DOI:** 10.1371/journal.pone.0295789

**Published:** 2023-12-14

**Authors:** Hamed Keramati, Adelaide de Vecchi, Ronak Rajani, Steven A. Niederer

**Affiliations:** 1 School of Bioengineering and Imaging Sciences, King’s College London, London, United Kingdom; 2 National Heart and Lung Institute, Imperial College London, London, United Kingdom; 3 Cardiology Department, Guy’s and St, Thomas’s Hospital, London, United Kingdom; 4 Turing Research and Innovation Cluster in Digital Twins (TRIC: DT), The Alan Turing Institute, London, United Kingdom; NED University of Engineering and Technology, PAKISTAN

## Abstract

Accurate velocity reconstruction is essential for assessing coronary artery disease. We propose a Gaussian process method to reconstruct the velocity profile using the sparse data of the positron emission particle tracking (PEPT) in a biological environment, which allows the measurement of tracer particle velocity to infer fluid velocity fields. We investigated the influence of tracer particle quantity and detection time interval on flow reconstruction accuracy. Three models were used to represent different levels of stenosis and anatomical complexity: a narrowed straight tube, an idealized coronary bifurcation with stenosis, and patient-specific coronary arteries with a stenotic left circumflex artery. Computational fluid dynamics (CFD), particle tracking, and the Gaussian process of kriging were employed to simulate and reconstruct the pulsatile flow field. The study examined the error and uncertainty in velocity profile reconstruction after stenosis by comparing particle-derived flow velocity with the CFD solution. Using 600 particles (15 batches of 40 particles) released in the main coronary artery, the time-averaged error in velocity reconstruction ranged from 13.4% (no occlusion) to 161% (70% occlusion) in patient-specific anatomy. The error in maximum cross-sectional velocity at peak flow was consistently below 10% in all cases. PEPT and kriging tended to overestimate area-averaged velocity in higher occlusion cases but accurately predicted maximum cross-sectional velocity, particularly at peak flow. Kriging was shown to be useful to estimate the maximum velocity after the stenosis in the absence of negative near-wall velocity.

## Introduction

Blood flow reconstruction based on measured data in coronary arteries is crucial to calculate the measures used for estimating the severity of coronary artery disease. Using quantified velocity and pressure distributions guides clinicians to identify the exact location of the lesion and the suitable treatment procedure. Invasively, one can measure the pressure difference along a lesion during coronary angiography [1] to calculate the Fractional Flow Reserve (FFR), which links to the severity of the disease. To avoid invasive procedures, techniques such as coronary computed tomography (CT) [2,3], 4D MRI [4,5], and cardiac Positron Emission Tomography (PET) [6] have been used. However, all the current techniques are associated with various uncertainties and challenges.

Positron Emission Particle Tracking (PEPT) is a technique that enables us to track tracer particles in opaque environments [7] with novel medical applications [8–11]. PEPT is based on PET and benefits from high spatial and temporal resolution [12].

Tracer particles are labeled with positron emission radionuclides such as ^18^F, ^61^Cu, ^89^Zr, and ^66^Ga [12,13] and then the particles are injected into the system. When the particles are in the field of view, the positron-emitting traces are tracked, and the trajectories are reconstructed using the gamma rays detected by the gamma cameras and corresponding lines of response (LORs). PEPT particle tracking methods started with the Birmingham method [14,15], and have been expanded to include the line-density method [16], multiple location-allocation algorithm (MLAA) [17,18], K-Medoids [19], clustering methods [20], the feature point identification (FPI) method [21], Odo triangulation method [22], Voronoi-based multiple particle tracking (VMPT) [23], the time-of-flight PEPT (TOF-PEPT) algorithm to do motion correction in medical imaging [24,25] and recently-developed method of PEPT machine learning (PEPT-ML) which tracks multiple particles and does not require frame tracking [26].

PEPT was initially used in industrial applications with opaque and fast-moving environments such as industrial powder mixing systems [27] and complex deformation such as extrusion [28,29]. PEPT has also been used for the characterization and measurement of physical behaviors such as diffusion [30] and convection [31]. However, PEPTs ability to track fluid flow in opaque systems has yet to be widely exploited in medical applications.

To accelerate the development of flow reconstruction methods and optimize PEPT experimental designs, Computational Fluid Dynamics (CFD) is advantageous. CFD can encode inherent physical constraints to reconstruct flow fields to distinguish the effects of various sources of uncertainties in this novel technology. Previously, CFD-PEPT studies have focused on industrial applications. The location of tracers in turbulent flow was predicted in a 3D tube with an obstacle using PEPT and Large Eddy Simulation (LES) models [32]. Experimental investigations were also performed to trace a single particle moving through a bend in a pneumatic system [33], in a hydro-cyclone with a turbulent flow for industrial applications [34], and in a tube with a pulsatile flow [35]. PEPT was also used as an experimental validation for a granular dynamics model coupled with CFD [36].

Recently, PEPT has been adopted for biomedical research and applications. Such applications include the improvement of radiation therapy [37] and tracking cells in the body [8,11].

For the clinical application of PEPT, the main advantage is that it enables us to track particles *in vivo*, i.e., inside the patient’s body [9]. In contrast to conventional particle tracking imaging techniques, e.g., PIV, PEPT does not require a transparent environment. The second advantage is that PEPT uses a PET scanner to collect the particle data. By improving the PEPT technology, one can use the existing and standard PET scanner to image and diagnose new conditions.

By injecting the tracer in the blood flow upstream of the location of interest, using PEPT one can relate the blood velocity to the measured tracing particles and can estimate the blood velocity in different locations in vessels. Using PEPT enables researchers and clinicians to reconstruct the velocity profile. This can be used to quantify other metrics of diagnostic relevance, such as the wall shear stress [38], which is related to the risk of thrombus formation [39,40]. Moreover, the velocity profile after the stenosis can be used to estimate the pressure drop across the blockage without catheterization [41].

Assuming the surface forces between the particles, the effect of the blood rheology, and the interaction between the particles and the blood cells are negligible, the low Stokes number reported by [33] implies the path of the particles in the flow is only governed by the velocity field of the flow, not the particle inertia, meaning the effect of the particles on the flow is negligible. Bruggemann and co-workers [42] used the Monte Carlo technique implemented in the package GEANT4 Applications for Tomographic Emission (GATE) [11] to reconstruct the flow downstream of an orifice to find the required acquisition duration to resolve the flow features of interest for a given activity and the Reynolds number for simulated PET data. GATE was also used to assess the ability of PEPT to track particles in turbulent flows [43] and to model a PET scanner to for the for the development of new PEPT algorithms [44]. Clinical CFD-PET simulations have been reported in [45] to predict the radioisotope distribution in the hepatic artery during a dosimetry procedure to optimize the injection site for tumor targeting. Although they did not perform particle tracking analysis, they observed that the number of microspheres was proportional to the cumulative blood flow.

One of the sources of uncertainties in reconstructing the velocity profiles is the sparsity of the particle data, especially in a time-dependent flow. To overcome this challenge, we proposed and evaluated the kriging Gaussian process to calculate the blood flow velocity based on the velocities of the particles and to quantify the uncertainty based on the sampled data. Kriging, an advanced geostatistical technique, has emerged as a powerful tool in various fields, including geology [46], design optimization [47–49], health-related data analysis [50–54], and medical image processing [55–57]. Kriging models complex spatial patterns and incorporates spatial dependency, offering an innovative solution for capturing and predicting data in areas where traditional interpolation methods fail [40]. The model is trained using the measured or sampled data, and the kernel hyperparameters are optimized. By utilizing a Gaussian process and a set of predefined parameters, kriging not only estimates unknown values at unsampled locations but also provides measures of uncertainty, enabling researchers to make informed decisions based on the reliability of predictions.

So far, PEPT has mainly been used to record the position of a single particle. However, to reconstruct the velocity profile of blood flow with the accuracy required for diagnostic purposes, more data points are required. In addition to the number of particles, other factors like the number of seeding instances in a cardiac cycle play a central role in the accuracy of the velocity reconstruction. Therefore, we investigated the effect of the number of particles and the measurement sampling rate per cardiac cycle on the uncertainty associated with the velocity reconstruction and quantified the error in velocity field estimates in a set of models with increasing levels of complexity.

In this paper, we present a fully computational methodology for blood flow reconstruction from multiple particles to assess the potential of clinical applications of PEPT for coronary disease diagnosis and estimation of the associated uncertainty. Specifically, we apply Gaussian process to estimate blood velocity and the associated uncertainty, first in an idealized straight vessel with stenosis, then in a bifurcation with a stenotic branch, and finally in a patient-specific model of coronary obstruction.

The current paper presents a fully *in-silico* study, which is a necessary step to inform our next steps for in-vitro and finally *in-vivo* experiments. These controlled in-silico datasets form a testbed to understand the feasibility of PEPT and Gaussian process in *in-vitro* phantoms of arterial flow. Full *in-silico* approaches improve one’s understanding of the strength and weakness of the reconstruction algorithm and are beneficial for designing *in-vitro* and *in-vivo* experiments.

## Methods

In our test scenario, pulses of biocompatible tracer particles [10,58] are injected into the patient upstream of the region of interest. Sufficient particles in the plane of interest in the vessel are needed to reconstruct the velocity field. If only one particle in the region is used, it is not possible to calculate the mean velocity as the true velocity profile is not known, and because of this, we have increased the number of tracers.

**Fig 1:**
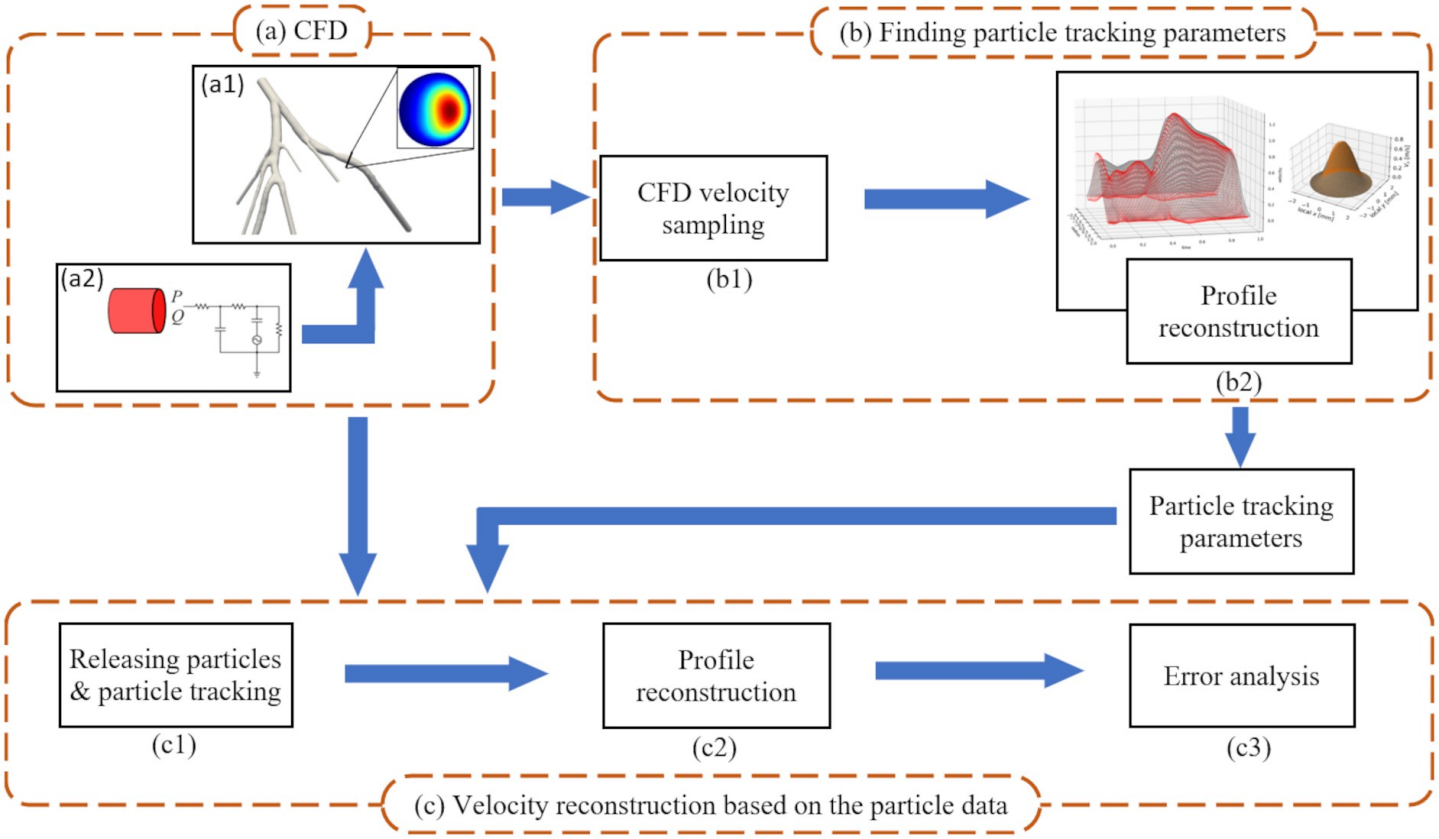
Schematic of the research methodology. The velocity field is generated using CFD (a). By sampling from the CFD velocity field, the number of required particles for particle tracking is estimated (b). The particle tracking is performed as a post-processing procedure and the velocity profiles are reconstructed (c).

Fig 1 shows the workflow for the present analysis. CFD simulations are first performed to generate a physiological flow field (a), from which random points are iteratively sampled at a specific location to reconstruct the profile in space and time (a1). An error analysis is then performed to establish the optimal number of sampling points necessary to reconstruct the velocity field (a2). We then determine the optimal number of simulated PEPT particles that need to be released based on the optimal number of sampling points from the error analysis in the CFD model. At the next step, we simulate particle tracking to approximate the PEPT procedure (c1). The velocity profile is then reconstructed using kriging and the particles’ velocity as they pass through a plane of interest (c2). Finally, the reconstructed velocity field is quantitatively compared with the velocity field from CFD (c3).

### CFD simulation

We propose a workbench of CFD problems for evaluating PEPT spanning from a straight tube to a bifurcation and finally a patient specific coronary obstruction model.

#### Computational domains

**Fig 2:**
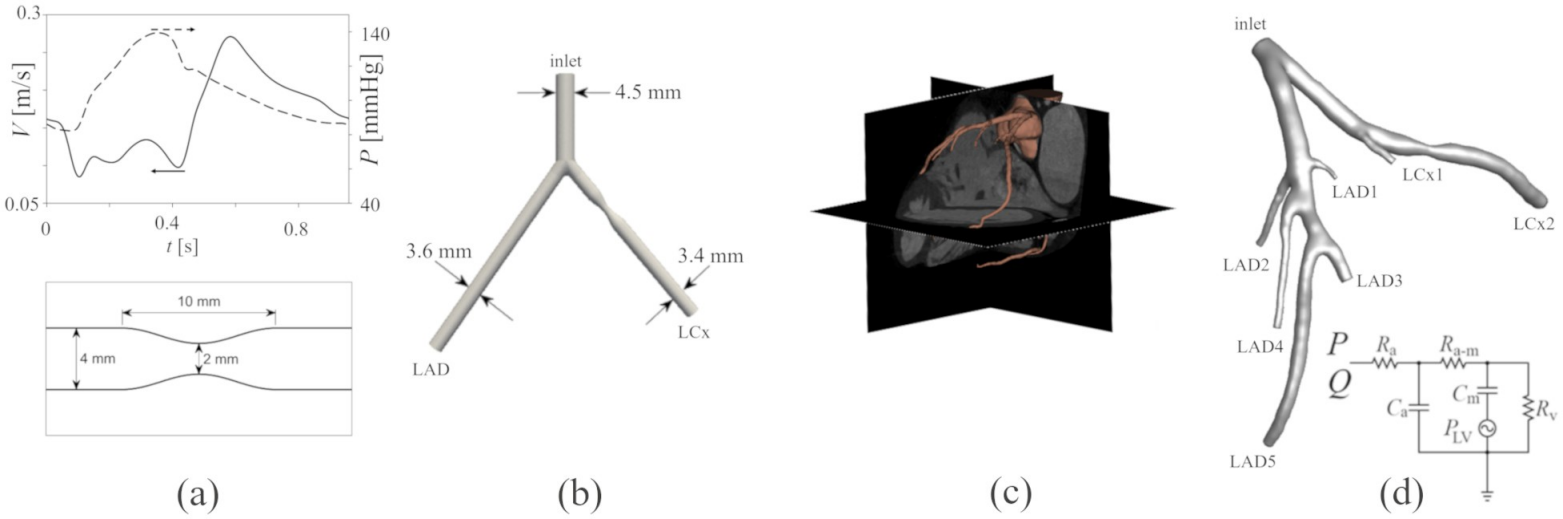
Geometry and boundary conditions. Tube stenosis geometry, the stenosis geometry, velocity (solid line) and the pressure (dashed line) regenerated from [59] (a), idealized bifurcation with 50% stenosis at LCx (b), segmented coronary artery on the CT scan images (c) and anatomical bifurcation with an artificially generated 50% occlusion at LCx and the equivalent electrical circuit for the outlet boundaries (d).

For the simplest model we considered a straight tube with a 50% blockage (Fig 2a), where the occlusion profile was generated using a sinusoidal shape. Both inlet and outlet boundaries were extended by 60 mm.

For the idealized bifurcation model (Fig 2b), the diameters and lengths of the vessels were based on previous studies on coronary flow [60,61]. Murray’s law was used to relate the radii of the branches to the radius of the main inlet, as well as the angles between each branch and the centerline, which are 35° and 40° for the left anterior descending artery (LAD) and the left circumflex (LCx), respectively [62]. In each case, models with a narrowing of 30%, 50% and 70% in the cross-sectional diameter were created. Finally, the anatomy of a patient-specific coronary tree (Fig 2c) was reconstructed from anonymized cardiac CT images using 3D Slicer 4 [63] and MeshMixer (Autodesk, California, USA). The computational domain is presented in Fig 2d.

#### CFD simulation and boundary conditions

The Navier-Stokes and continuity equations for incompressible flows were solved using Ansys^®^ FLUENT^®^, Release 18.1 (ANSYS, Canonsburg, PA, USA). Regarding the Reynolds number for the average velocity (Re<500) the flow was assumed to be laminar [64]. Even though Re at the peak flow in a stenosis might reach transition values, the pulsatility effect means that turbulence will not develop at the scale that requires turbulent modeling. The blood was assumed to be a Newtonian flow with a density of 1050 kg/m3 and a viscosity of 0.0036 Pa∙s. According to [65], assuming the blood to be Newtonian is acceptable in coronary artery, which leads to more computationally efficient simulations. The velocity waveform recorded in 20 patients with relatively low probability of coronary disease [59] (Fig 2a) was prescribed as the inlet boundary condition (BC). For the straight tube, the outlet pressure presented in Fig 2a [59] was used as the outlet BC. For the idealized bifurcation geometry and the patient-specific anatomy, a 0D model was assigned to the outlet boundaries based on the model described by [66] as shown in Fig 2d. The equation for the 0D model is:

$$a\frac{d^{2}P}{dt^{2}}+b\frac{dP}{dt}+cP=d\frac{d^{2}Q}{dt^{2}}+e\frac{dQ}{dt}+fQ+g,$$ (1)

where *P* is the pressure and *Q* is the flow rate. The rest of the parameters are as follow:

$$\begin{array}{l} a=R_{\text{v}}C_{\text{im}}R_{\text{a}-\text{m}}C_{\text{a}},\\ b=R_{\text{v}}C_{\text{im}}+R_{\text{v}}C_{\text{a}}+R_{\text{a}-\text{m}}C_{\text{a}},\\ c=1,\\ d=R_{\text{a}-\text{m}}R_{\text{a}}R_{\text{v}}C_{\text{im}}C_{\text{a}},\\ e=R_{\text{a}}R_{\text{v}}C_{\text{im}}+R_{\text{a}-\text{m}}R_{\text{v}}C_{\text{im}}+C_{\text{a}}R_{\text{a}}R_{\text{v}}+R_{\text{a}-\text{m}}R_{\text{a}}C_{\text{a}},\\ f=R_{\text{a}-\text{m}}+R_{\text{a}}+R_{\text{v}},\\ g=R_{\text{v}}C_{\text{im}}\frac{\text{d}P_{LV}}{\text{d}t}, \end{array}$$

where *P*_LV_ is the pressure of the left ventricle and the lumped parameters for the resistances and capacitances *R*_a_, *R*_v_, *R*_a-m_, *C*_im_, and *C*_a_ are illustrated in Fig 2d. The lumped parameters were then separately calibrated in both geometries based on physiological flow curves [59]. The parameter optimization was performed using Simulink^®^ (MathWorks, Natick, MA, USA) by minimizing the error between the physiological inlet pressure [59] and the calculated inlet pressure. The final parameter values are listed in S1 and S2 Tables for idealized bifurcation and anatomically-accurate geometry, respectively. The CFD simulation was performed for three cardiac cycles until the solution reached a periodic state.

We carried out a grid independence analysis to ensure the results were in the asymptotic region. The used mesh for all stenotic cases had 800k to 831k cells. The error between the area-pressures was less than 1.5% and the maximum velocity in the field had 0.7% error when the mesh was refined about 50%.

We ensured that the pressure is within a physiological range (70–140 mmHg), allowing us to compare simulated PEPT particle distributions within under physiologically plausible conditions [67].

#### Flow measurement

To estimate the flow across an occlusion, particles were released randomly in a cross section at the inlet of the vessel. The location at which each particle experienced its maximum velocity was then used to estimate the position of the occlusion. The particles’ velocity through a plane of interest (POI) perpendicular to the vessel centerline 10 mm downstream of the minimum lumen area (MLA) was then calculated. This location was chosen based on the guideline for the Fractional Flow Reserve (FFR) measurements [68,69]. The reconstructed profile, performed using kriging, was compared with the results from CFD.

### Sampling

The velocity vector from the CFD solution was sampled at a varying number of points uniformly distributed in the POI (i.e., 10, 20, 40, 60, 80 and 100). *T*_c_ is the cardiac cycle time (*T*_c_ = 0.96 s), *t*_c_ ∈ [0,*T*_c_] is the time in a cycle, and Δ*t*_c_ is the difference between the *t*_c_ values. The data were sampled at Δ*t*_c_ values of 96 ms, 64 ms and 48 ms, equivalent to sampling 10, 15 and 20 times per cardiac cycle. Δ*t*_c_ was considered as a control parameter in this study.

Sampling allowed us to find the optimal number of particles and Δ*t*_c_ for the particle tracking algorithm (Fig 1b). In the particle tracking phase, Δ*t*_c_ can be interpreted as the time points at which the particles are injected within the cardiac cycle.

### Particle tracking

The Stokes number of a particle in a flow represents the time it needs to follow the changes in the flow field normalized by the characteristic time of the flow, i.e., the ratio of the particle relaxation time, *τ*_p_, and the flow typical time scale, *τ*_f_ [70]:

$$St=\frac{\tau_{p}}{\tau_{f}}.$$ (2)

The characteristic time of a particle is defined [71] as

$$\tau_{p}=\frac{\rho_{p}d_{p}^{2}}{18\mu_{f}},$$ (3)

where *ρ*_p_, *d*_p_ and *μ*_f_ are the density of the particle material, the particle diameter and the surrounding fluid viscosity. For a particle in range of 1–100 μm [12] and with density similar to that of blood, *τ*_p_ is between 1.62 × 10^−8^ s and 1.62 × 10^−4^ s. Therefore, for *τ*_f_ of about a second in the coronary artery, the Stokes number will be less than 10^−3^, which means that the particles are driven by the flow without disturbing it. The velocity of a particle at a point can therefore be approximated by the velocity of the carrier fluid at the same point and the particle tracking analysis can be de-coupled from the flow simulation. Based on these considerations, particles were released in the computed flow field before the stenosis and tracked by calculating their location at the next particle tracking time step (Δt_PT_) using in-house software. The updated location of the particles at the time k+1 was calculated as:

$$x^{k+1}=x^{k}+v{\Delta t}_{PT}+\frac{1}{2}a\Delta t_{PT}^{2},$$ (4)

where **v** and **a** are the velocity and acceleration vectors from the CFD simulation. Both **v** and **a** depend on space and time and should be updated at each time step. Where historical information was lacking (for *k* = 0, 1), the acceleration was assumed to be zero. Algorithm 1 presents the step by stem algorithm.

**Algorithm 1** Post processing particle tracking

 Load CFD results for all time steps

 Initialize the location **x** of the tracer particles

 Initial the time *t* = *0*

 **while** *t* < *t*_end_ **do**

  Check all elements of the particle location to be in the domain

   **if** no element located in the domain

    **end procedure**

  Read the velocities at **x** and *t*

  Calculate the acceleration

   **if** *t* = 0 or Δ*t*_PT_ **then**

    Acceleration is zero **a** = 0

   **else**

    Linear acceleration based on the previous velocities

  Update *t* to *t* + Δ*t*_PT_

  Update **x** using Eq 4

 **end while**

Similar to the sampling phase, we grouped the data points with similar Δ*t*_c_. Therefore, although the particles could be injected in different cardiac cycles, we grouped the information of the particles that have identical *t*_c_ values. As for the CFD sampling, the quantity Δ*t*_c_ was also considered as a control parameter for the particle tracking. It should be emphasized that Δ*t*_c_ is a property of seeding/grouping the particles during the analysis of the results.

The assumption of multiple particles was used because the velocity depends on both space and time, therefore using multiple particles is necessary for generating enough data to train the Gaussian process.

It is worth noting that we initially assumed perfect tracking, i.e., the location of the location of the particles were exactly measured and the positron range was assumed to be zero. However, the positron range in a blood-like environment is in submillimeter scales for ^18^F [72]. This range affects the spatial resolution of PEPT. We initially assume the limiting case, where we decay events occur at a sufficient rate that allow an accurate partial position to be determined through averaging. To estimate the impact of uncertainty on our analysis we consider the effect of noise. We have introduced random noise to the location of particles before feeding them to the kriging process. Moreover, this approach provides an estimate of what can be achieved at given levels of uncertainty and provides an estimate of the accuracy upper bound that could be achieved with improved sensors that approached optimal data acquisition.

### Kriging

To reconstruct the velocity using the data from a limited number of particles, we used the kriging technique. In kriging, the points closer together have more similar values than points further apart. The values corresponding to the points are related through the semivariogram. For the current study, we used an anisotropic exponential semivariogram with a higher impact on the spatial correlation than the temporal correlation. In kriging, one uses the data value at some special points and calculates the continuous distribution of the parameter. During the training phase, the semivariogram (i.e., the kernel) was calculated based on the sampled data and the values for the hyper-parameters, i.e., sill, range and nugget were calculated. The nugget, i.e., the y-intercept for the semivarogram, was consistently zero for all cases in our analysis. Therefore, the kriged field behaved as an exact interpolator.

Regarding the pulsatile nature of the flow, we used universal kriging that does not assume the constant mean value for the data. The data for training the kriging process was prepared based on the locations and velocities of the sampled points (for the sampling) and the particle locations and the corresponding velocity (for the particle tracking). We transform the 3D coordinate system to local 2D coordinates at the POI. By doing so we transformed (*x*,*y*,*z*,*t*) into (*X*,*Y*,*t*) to use a 3D kriging process, where *x*, *y* and *z* were the global 3D coordinates for the data and *X* and *Y* were the local 2D corresponding coordinates on the POI. The time was not changed in the transformation. Zero velocity was set at the boundary of the POI for all time steps to apply the no-slip condition of the flow at the vessel wall. As the rigid wall was assumed for the CFD domain and the fluid was modelled as a continuous material, no-slip boundary condition was chosen for the study. The S1 Text presents the universal kriging process which was used for reconstructing the normal velocity profile.

#### Error analysis

In this study, we employed two concepts of error and uncertainty. The error refers to the difference between the CFD (ground-truth) and the reconstructed velocity profile. The reconstructed profile at each point is space and time is the mean of the value of the values predicted from the Gaussian process. On the other hand, the range that the Gaussian process predicts for a point with no particle or sample data is the quantified uncertainty. The uncertainty of the prediction is calculated during the Gaussian process and reflected in the kriging variance. The uncertainty is not compared to the true values from the CFD simulations.

We used two error measures to evaluate the accuracy of the kriging velocity reconstruction based on the sparse data from particles: the root-mean-square (RMS) error between the reconstructed profile and the true velocity profile from the CFD simulations, and the difference of the peak velocity value from the reconstructed profile and that from the CFD simulation. For the first error, the reconstructed velocities were compared to the CFD results and the RMS effective surface error, *e*_RMS_, was used to quantify accuracy [73]:

$$e_{RMS}=\sqrt{\frac{\int e^{2}dA}{A}},$$ (5)

where *e* is the absolute error between the reconstructed profile and the CFD profile and *A* is the area of the POI. The error was normalized with the average velocity at the corresponding timepoint.

For the maximum velocity error, *e*_max(*v*)_, the relative error of the maximum reconstructed velocities was calculated as:

$$e_{\text{max}\left(v\right)}=\frac{abs\left(\text{max}\left(v_{R}\right)-max(v_{CFD})\right)}{max(v_{CFD})},$$ (6)

where max(*v*_*R*_) is the maximum value of the reconstructed velocity normal to the POI at a given timepoint and max(*v*_CFD_) is the maximum value of the normal velocity from the CFD simulation at the same timepoint.

For more clarification, Table 1 presents all the errors used in this paper with their explanations.

**Table 1:** The errors and their explanations.

Error	explanation
*e*_RMS_	The root-mean-square (RMS) error between two 3D surfaces, e.g., velocity profiles
*e*_max(*v*)_	Relative error between the peaks of two surfaces, e.g., velocity profiles
*e*_par−CFD_	The normalized RMS error between the area-average velocity curves corresponding to the reconstructed velocity from the particle data and the true (CFD) results in a full cycle
*e*_sam−CFD_	The normalized RMS error between the area-average velocity curves corresponding to the reconstructed velocity from the sampled data and the true (CFD) results in a full cycle

## Results

### Stenosis detection

**Fig 3:**
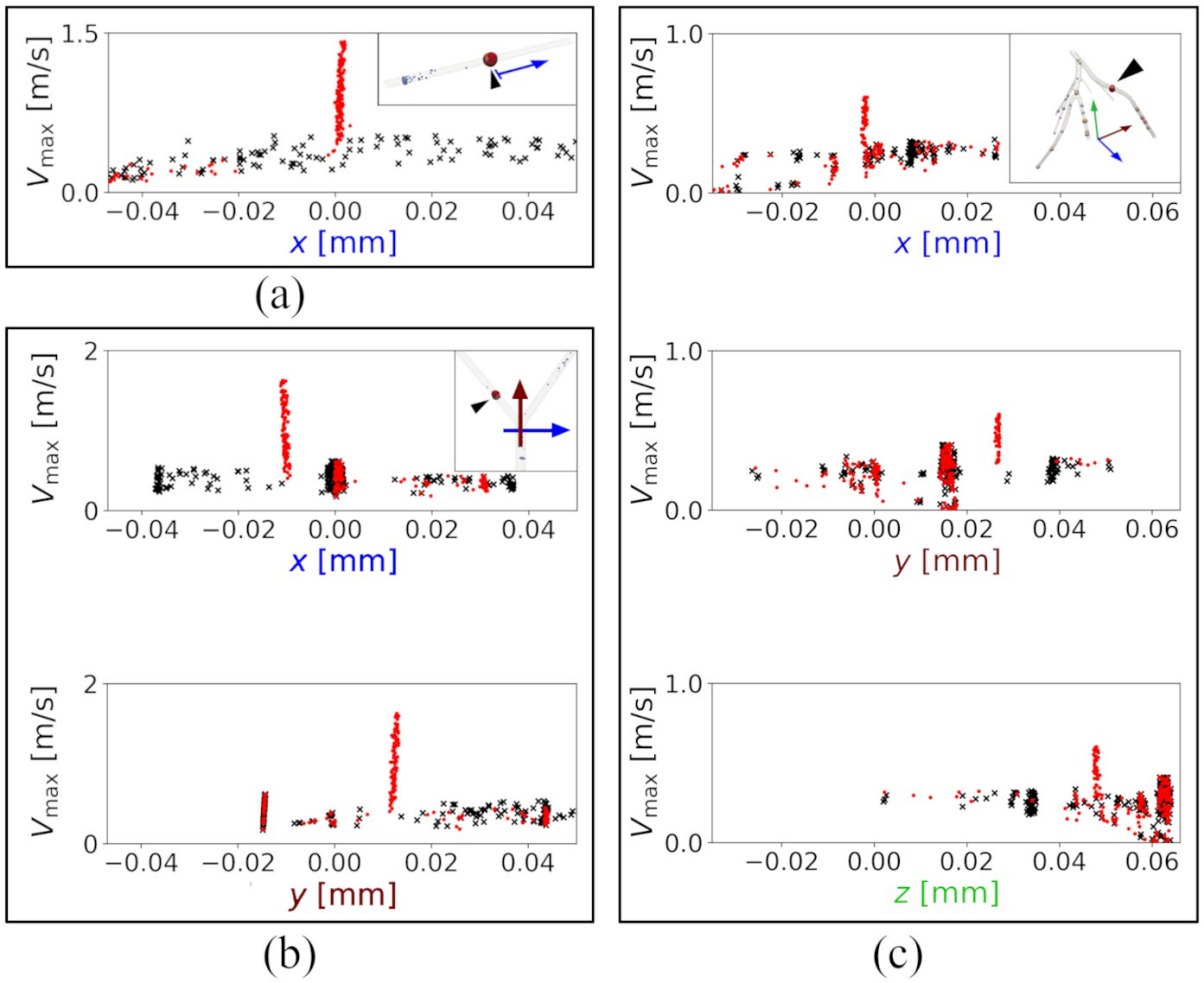
The maximum velocity of particles and the coordinates in which the maximum velocity occurred. Results in a straight tube (a), a bifurcation (b) and an anatomically accurate left coronary artery (c). Each point represents a particle. Points presented with black **×** correspond to the case without stenosis and the red dots ● correspond to the case with a 50% stenosis. The black triangle shows the location of the stenosis based on particle data in different geometries.

Fig 3 shows the maximum velocity and the corresponding position for each particle in the three geometries, with and without stenosis (red and black symbols, respectively).

The cycle time difference between the particle releases was Δ*t*_c_ = 64 ms and the particles were released in batches of 40.

The axial location of the stenosis was identified by the accumulation of particles with elevated maximum velocity. An illustration of the estimated Cartesian location of the stenosis based on the maximum velocity is presented in the embedded panel for each case.

### Sampling

**Fig 4:**
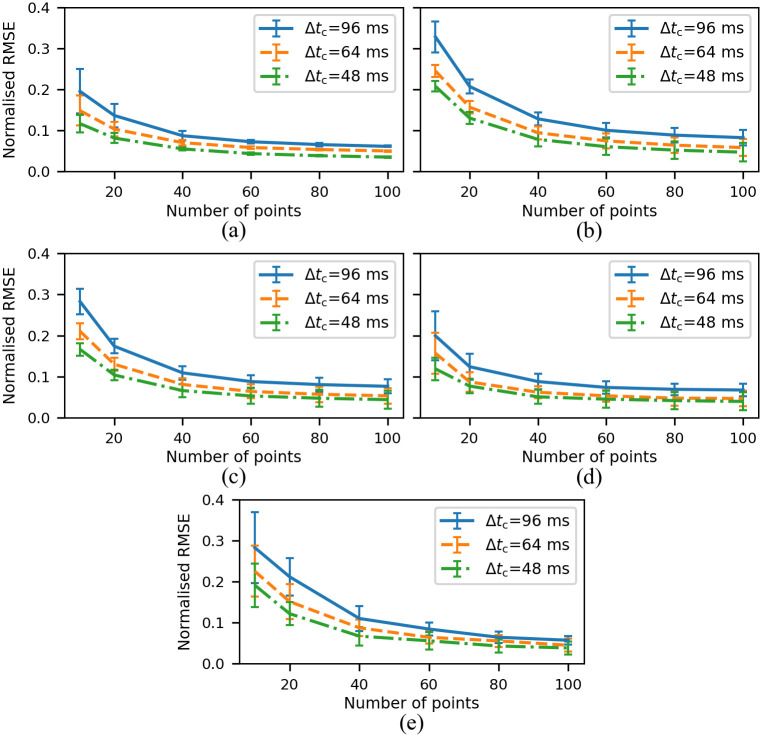
The error for the velocity reconstruction using the sampled points. The normalized RMS error of the area-averaged velocity over a cardiac cycle normalized with the CFD total averaged velocity from CFD for different number of sampling points and Δ*t*_c_ for a tube with 50% of occlusion (a), a bifurcation without occlusion (b) and a bifurcation with 30% of occlusion at LCx (c), a bifurcation with 50% of occlusion at LCx (d) and a bifurcation with 70% of occlusion at LCx (e). The error bars show the standard deviation. The cardiac cycle time, *T*_c_ is 0.96 s.

The normalized *e*_RMS_ in the area-averaged reconstructed velocity was found to decrease monotonically with increasing numbers of sampling incidents and points. This behavior is shown for the straight vessel with 50% occlusion (Fig 4a) and the bifurcation model with 0%, 30%, 50% and 70% occlusion (Fig 4b–4e). The error bars show the standard deviation calculated from 30 repeat simulations.

When 40 or more sampling points are used with Δ*t*_c_ of 64 ms or 48 ms (6.7% or 5% of the cycle), the median normalized *e*_RMS_ were less than 10%. For 10 sampling incidents, an error of less than 10% was achieved for 60 or more particles. The error standard deviations in the idealized bifurcation model with 70% occlusion (Fig 4e) are smaller than those of the cases with less severe occlusion (Fig 4b–4d).

By increasing the number of sampled points, the errors decreased from over 30% to less than 10% for the case without an occlusion and Δ*t*_c_ = 96 ms. Similar behavior was observed for all other cases.

The same error analysis was performed at POI for the time point at which the maximum flow rate occurred. The normalized RMS error showed a similar behavior to that presented in Fig 4, with values less than 10% for 40 or more sampling points and Δ*t*_c_ of 64 ms and 48 ms for all cases. To achieve convergence, we performed 30 trials of randomly sampled points at the POI.

### Particle tracking

#### Area-averaged velocity

To better approximate PEPT, we simulated the motion of particles in the flow. We compared the reconstructed peak velocity and the profile at the POI calculated from kriging and those from the CFD. Based on the error analysis, the particle tracking was performed in all models using 600 particles (15 batches of 40 particles) and Δ*t*_c_ = 64 ms. Convergence was achieved with 10 trials of tracking randomly released particles.

**Fig 5:**
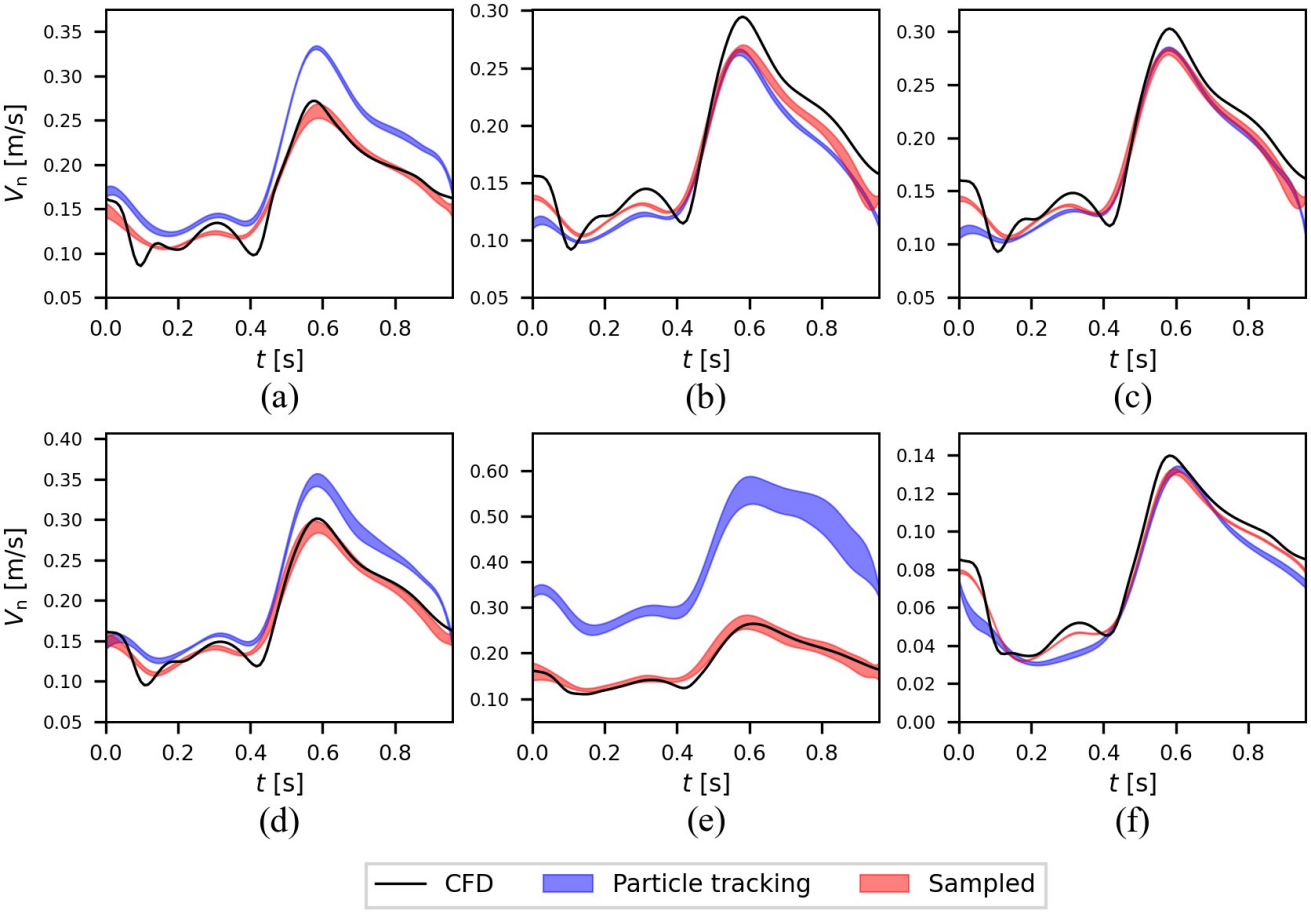
Comparison of the area-averaged velocity reconstructions. The area-averaged velocity against time for a straight tube with 50% occlusion (a), idealized bifurcation with 0% (b), 30% (c), 50% (d) and 70% (e) occlusion, and an anatomic coronary artery with 50% occlusion (f). All error values were normalized with the averaged CFD normal velocity over the cycle, and the shaded areas show the standard deviation based on the simulation repeats.

The area-averaged velocity against time for the CFD velocity profile, the reconstructed profile based on the sampled data, and the reconstructed profile based on the particle tracking data for all cases are presented in Fig 5.

For the flow in the straight tube with 50% occlusion, the area-averaged velocities at the POI from the CFD (ground truth) and both reconstructions from sampled points and particle tracking (with the same number of particles and seeding instances) show a consistent overestimation of the ground truth by the particle tracking algorithm (Fig 5a). The error between the particle tracking velocity reconstruction and the CFD, *e*_par−CFD_, and between the sampled velocity reconstruction and the CFD, *e*_sam−CFD_, were 29.9% and 6.6%, respectively, indicating that particle tracking introduced a significant increase in reconstruction error.

Fig 5b–5e, present the time-dependent area-averaged normal velocity at the POI for the idealized bifurcation with 0%, 30%, 50%, and 70% occlusion at LCx. We calculated the RMS error between the area-averaged velocity curves corresponding to particle tracking and CFD in Fig 5 and normalized that with the time mean CFD velocity over a cycle. While the normalized RMS error for the sampling procedure remained under 10% for all cases, the error for the particle tracking was 10.2% for 30% occlusion (Fig 5c), 16.9% for 50% occlusion (Fig 5d) and 128.1% for 70% occlusion (Fig 5e).

Fig 5f shows the average velocities for the anatomically accurate model with 50% occlusion. The normalized RMS errors, i.e., *e*_par−CFD_ and *e*_sam−CFD_, were 15.1% and 6.2%, respectively. None of the reconstructed velocity profiles could replicate the oscillatory behavior of the flow between 0 and 0.4 s. The results are summarized in Table 2.

**Table 2:** The error between the particle tracking velocity reconstruction and the CFD, *e*_*par*−CFD_, and between the sampled velocity reconstruction and the CFD, *e*_*par*−CFD_, for different cases.

Geometry	Straight tube	Idealized bifurcation	Idealized bifurcation	Idealized bifurcation	Anatomical geometry
Occlusion [%]	50	30	50	70	50
*e*_par−CFD_	29.9	10.2	16.9	128.1	15.1
*e*_sam−CFD_	6.6	7.9	5.4	5.9	6.2

#### Error analysis

We compared the accuracy of the kriging velocity profile reconstruction based on the particle tracking by calculating RMS errors between the CFD velocity profile and the reconstructed profiles. Table 3 shows the different types of RMS errors between the reconstructed velocity profile and the CFD velocity profile (Eq 5), i.e., the maximum value, the value at the peak flow and the mean value over the cycle for the normalized *e*_RMS_.

**Table 3:** The normalized RMS error for the profile reconstruction by the particle tracking data ± the standard deviation over random trials. The error is normalized with the averaged normal velocity at the same time point.

Geometry	Occlusion [%]	max(*e*_RMS_) [%]	*e*_RMS_ @ of peak flow [%]	mean(*e*_RMS_) over a cycle[%]
Straight tube	50	73.8±3.6	33.8±0.5	33.6±0.7
Idealised bifurcation	0	31.3±3.2	16.3±0.4	13.4±1.3
Idealised bifurcation	30	42.7 ±4.1	8.4±1.0	13.4±0.5
Idealised bifurcation	50	55.6±3.9	26.4±2.6	28.5±2.2
Idealised bifurcation	70	209.8±21.4	138.1±13.5	161.0±13.0
Anatomical geometry	50	54.4±3.0	12.5±1.0	21.9±0.9

Table 4 shows the maximum, minimum and time-averaged errors over a cardiac cycle for the estimation of the peak velocity at each time point (Eq 6).

**Table 4:** The relative error for maximum velocity prediction *e*_*max*(*v*)_ ± the standard deviation over random trials for particle tracking and sampling velocity reconstruction.

Geometry	Occlusion [%]	max(*e*_max(*v*)_) [%]	min(*e*_max(*v*)_) [%]	mean(*e*_max(*v*)_) over a cycle [%]
Straight tube	50	29.4±4.0	0.0±0.0	5.2±0.4
Idealized bifurcation	0	25.3±6.3	0.2±0.2	6.2±1.2
Idealized bifurcation	30	32.3±3.6	0.0±0.0	5.2±0.3
Idealized bifurcation	50	22.3±3.5	0.1±0.1	5.0±0.5
Idealized bifurcation	70	18.1±2.7	0.0±0.0	3.6±0.3
Anatomical geometry	50	31.0±8.2	0.2±0.2	9.4±1.3

Calculated errors show that PEPT measurement after the stenosis results in overestimating the area-averaged velocity (i.e., the flow rate); however, this method is capable to measure the maximum velocity in the POI with a time-averaged error of less than 10%.

It should be noted that the results in Table 4 show that using PEPT and kriging could result in high error for estimating the peak cross-sectional velocity, i.e., max(*e*_max(*v*)_). However, the time-averaged error, mean(*e*_max(*v*)_) over a cycle, shows an error value of less than 10%.

#### Retrograde velocity

**Fig 6:**
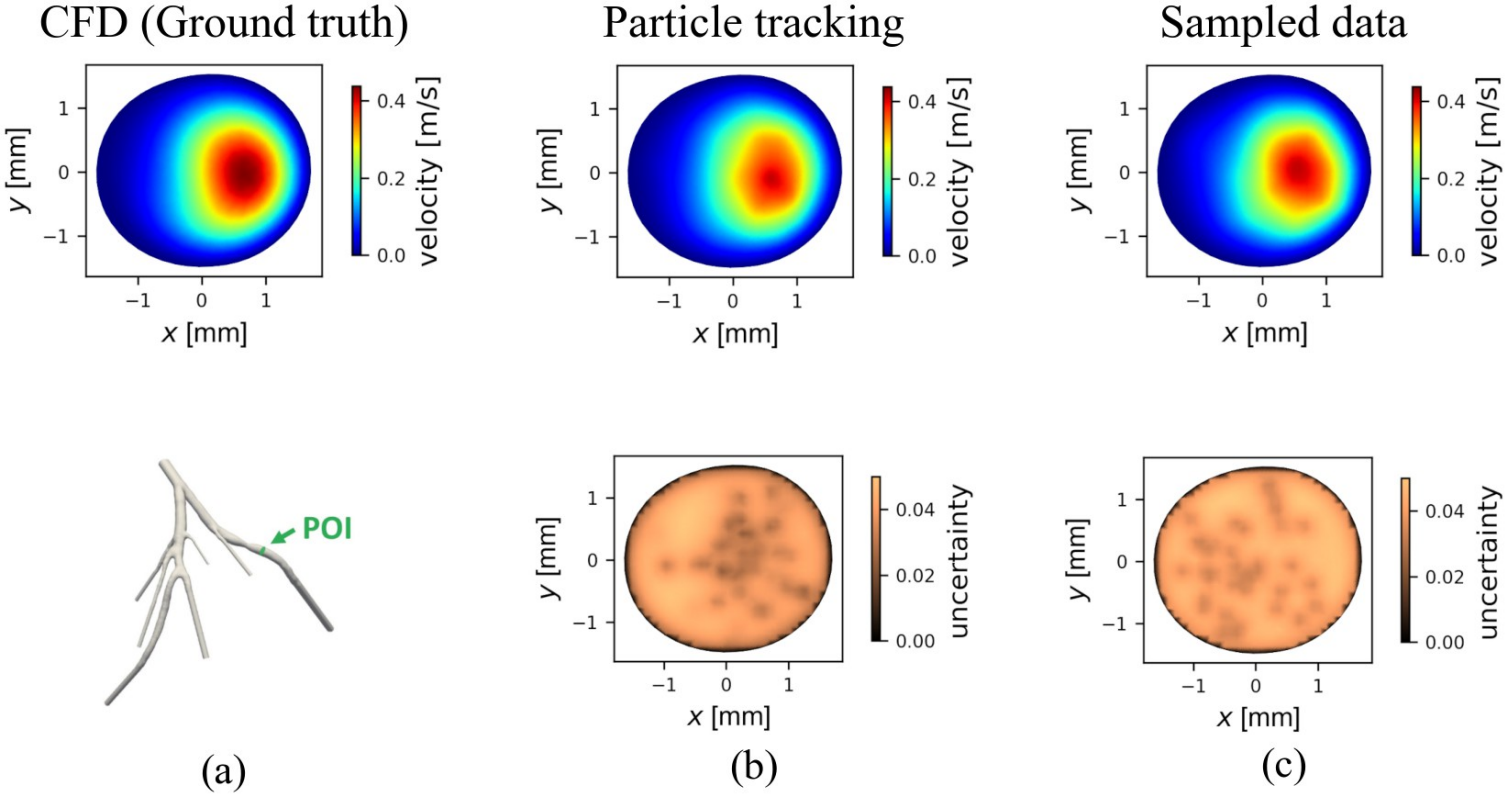
Velocity contours and the uncertainties. The normal velocity contour at the POI for the CFD simulation (a-top), the anatomy of the coronary artery (a-bottom), the velocity contour of the reconstructed velocity using the particle tracking data (b-top) and the local uncertainty (standard deviation for the velocity) calculated in the kriging Gaussian process (b-bottom), the velocity contour of the reconstructed profile using the sampling process data (c-top) and the corresponding local uncertainty (c-bottom). All the results are presented at the time when the peak flow happens (*t* = 0.582 s).

Fig 6 shows the anatomy, reference normal velocity contours, velocity reconstructed from sampling at the POI and velocity reconstructed from particle tracking, and kriging local uncertainties (standard deviations) for *t* = 0.582 s at which the maximum flow rate occurred for samples and particle-based velocity reconstruction. The figure illustrates the results for the CFD and the reconstructed profiles for the patient-specific case.

For both Fig 6b-bottom and Fig 6c-bottom the seeded particle and the sampled points were 40 and Δ*t*_c_ = 64 ms, respectively. In the kriging local standard deviation contour, the areas with lower uncertainty values were in close proximity to the sample points or where particles passed through the POI. As shown in Fig 6b-bottom, the uncertainty contour for the particle tracking, the area with lower uncertainty were clustered around the center of the artery, where we saw a higher density of particles. In contrast, Fig 6c-bottom uncertainty contour shows that in the sampling process, the distribution of the data points was more uniform. To estimate particle clustering, we calculated the standard deviation of the distance between all datapoints and their centroids for the particle tracking and sampling cases, *C*_d_ = SD(*d*_p_), where *d*_p_ is the vector of the distances between the points/particles and their centroid. At the POI, *C*_d_ for multiple random trials was 0.77±0.03 mm and 0.89±0.04 mm, for the particle tracking (10 random trials) and sampling case (30 random trials), respectively. The clustering causes a systematic increase in error in the velocity profile reconstruction near the borders.

**Fig 7:**
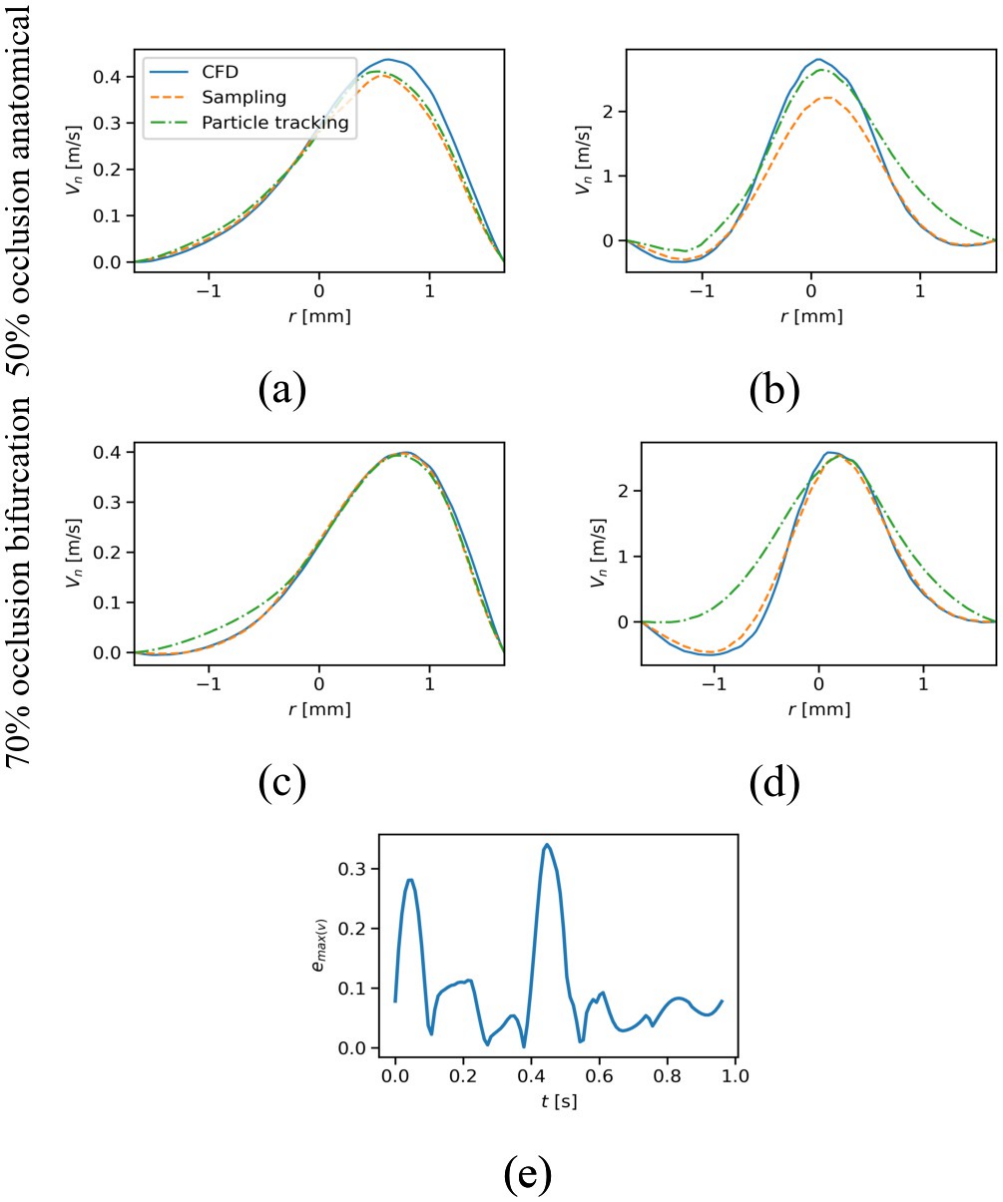
The reconstructed profile velocities and the error. The cross section of the velocity profiles of one of the simulation repeats, i.e., one of the trials for the particle tracking and random sampling, for the anatomically accurate case (50% occlusion) at *t* = 0.582 s (a) and *t* = 0.679 s (b) and the velocity profiles for the idealized bifurcation with 70% of occlusion at *t* = 0.582 s (c) and *t* = 0.679 s (d). Panel (e) shows the relative error for the maximum cross-sectional velocity during a cardiac cycle for the same simulation repeat.

To illustrate the difference between the particle tracking and the sampling profiles, Fig 7 shows cross-sections of velocity profiles for the same simulation repeats of Fig 6 and the corresponding errors.

Fig 7 shows an example for the local behavior of the kriging reconstruction procedure using the particle tracking data. As one can see, for cases without a negative velocity (Fig 7a) the kriging using particle tracking and sampling behaved similarly. For cases with larger negative velocity (Fig 7d) particle tracking failed to reconstruct the velocity. One should note that not all time points have particle data; therefore, the discrepancy between the particle tracking reconstructed velocity in Fig 7b and 7d in not only associated with the difference between the magnitude of the negative velocities but is also associated with the temporal and spatial distance between any point and the particle data.

#### Effect of noise

**Fig 8:**
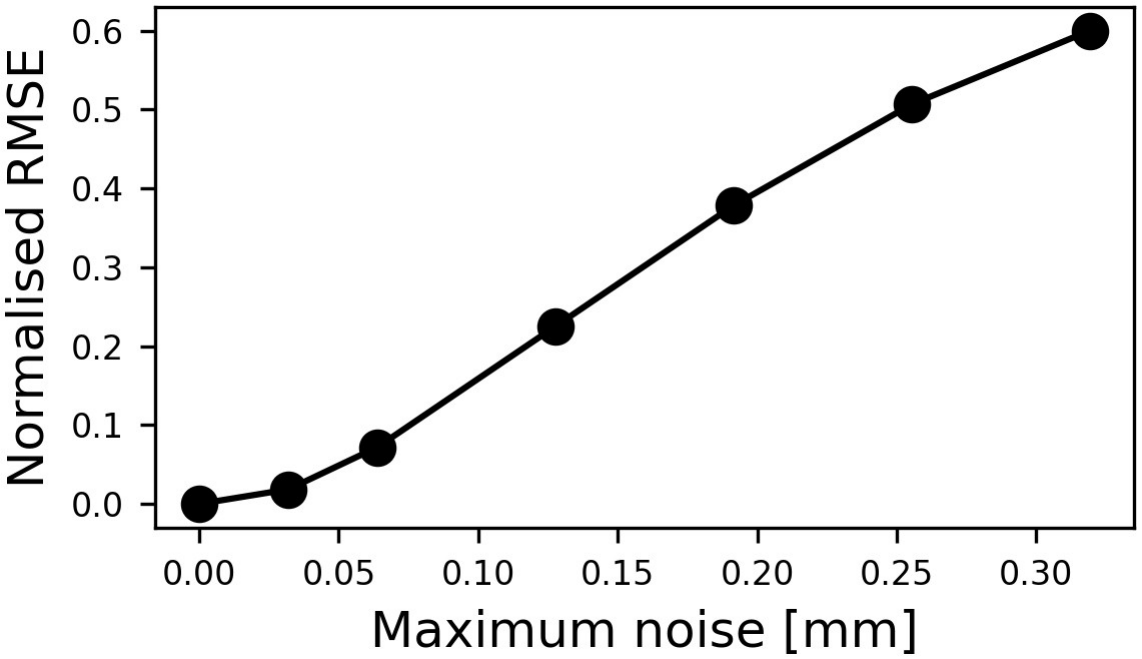
The effect of noise. The RMS error for the averaged velocity over the cycle for various maximum noise. The error was normalized based on the averaged velocity over the cycle.

To investigate the effect of the other sources of error, we added random noise to the particle location for the flow in the anatomically accurate case. In our study, we used independent noise in three directions for the particle positions in each time step. The random noise was generated with $X_{n}=N(0,I)$, where N is the normal distribution, and **I** is an identity matrix. Then, for different noise levels, *L*_n_, the noise, **X**_n_, was scaled in [−*L*_n_*r*, *L*_n_*r*] and added to the particle positions, where *r* is the radius of the cross section at POI before kriging. We have introduced an uncertainty less than 0.5 mm, regarding the scale of the coronary artery, on the location of the particles. Fig 8 shows the normalized RMS error against the area averaged velocity over a cardiac cycle for different maximum noise, i.e., *L*_n_*r*.

## Discussion

The motivation for this study is the novel medical application of PEPT. To develop and use PEPT for medical imaging and diagnosis of coronary artery disease, we took a computational approach to analyze the underlying errors of the reconstruction technique. We presented a methodology to reconstruct the velocity profile after an occlusion, which can then be used to calculate the wall shear stress [38,74,75] or estimate pressure gradients across the stenosis using Bernoulli equation [76] or the work-energy principles [41,77]. Our approach provides an estimated velocity profile in addition to the Lagrangian representation of the fluid flow as reported in [78]. This would allow PEPT to measure both blood velocity and functional pressure drop over an occlusion, in contrast to conventional PET, which can only measure perfusion over tissue volumes [79]. Cardiac 4D phase-contrast CMR can measure flow at a resolution of 1.5x1.5x1.5 to 3×3×3 mm^3^ [4,5] with a dependency on the users’ experience [80], which would limit their ability to measure flow across an occlusion.

To determine how many particles are required to estimate the velocity profile acceptably, we used sampling and then particle tracking to gather the velocity data from the flow field. However, the results show that an interpolation using particle tracking is less accurate than the profile derived from the direct sampling of the CFD results, especially when retrograde flow is present near the wall.

The virtual particles were seeded upstream of the blockage. Therefore, the particles were affected by any vortices that formed after the stenosis, provided that the CFD simulation could capture them. As the flow regime stays in the laminar region, a transient laminar CFD model is able to capture the vortices [64].

Using kriging and particle tracking allowed for an accuracy of 78.1% (mean RMS error over the cycle of 21.9%) for an anatomically accurate case. While these errors are high, they are comparable with alternate approaches. Reference [81] reported a novel method to estimate the average velocities in LAD using cine X-ray angiographic sequence (compared with transthoracic Doppler) with an average error of 30.8%±20% for 21 patients with no reported stenosis. Moreover, unlike [81], we did not need to make any assumptions regarding the diameter of the vessel while doing the particle tracking analysis, as PEPT can be performed on any vessel anatomy. The error values show that the kriging coupled with the particle tracking data can predict the maximum velocity at the POI for a given time more accurately (mean relative error of less than 10%).

We also demonstrated that peak particle velocity could be used to accurately identify the location of the stenosis. Due to the tendency of particles to cluster in the center of the vessel, where the peak velocity is higher, the reconstruction algorithm could detect the maximum velocity at peak flow with an error of less than 10% (Fig 7e). However, this also led to inaccuracy in the velocity profile reconstruction away from the centerline, where very few or no data points are available. This tendency is particularly exacerbated when areas of flow recirculation occur after a stenosis and cause negative velocity near the wall, which the kriging algorithm is unable to detect, resulting in large *e*_RMS_. Negative velocity in regions with the recirculating flow is due to the pulsatile nature of blood and the occlusion, which is consistent with the report from [42] that used simulated PET results to reconstruct the flow field in a jet flow and found a larger error in the recirculation region; however, the retrograde velocity in our study was always underestimated by the kriging and particle tracking. It is important to note that data from the radioisotope particles trapped in the recirculation region could result in errors due to the inability to differentiate the core flow and the recirculation flow. As shown in Fig 5a, 5d and 5e, the distribution of the particles around POI resulted in overprediction for cases with a high level of stenosis (50% and 70%). The higher inflow velocities in the idealized bifurcation compared to the patient-specific anatomy with a similar level of occlusion result in larger negative velocities at the POI and thus in larger errors in Fig 5e. Similar behavior was reported in [82] in biogas production and gas mixing applications about the existence of some regions in the domain that PEPT particles failed to enter and therefore higher uncertainties were calculated for the regions. As shown in Fig 7b, the particle tracking can recapitulate the flow velocity profile in the center of the vessel but fails to capture the flow profile at the edges of the vessel, where we see retrograde blood flow. As one can see in Fig 7c and 7d, the discrepancy between the particle tracking profile and the true profile is more severe in the bifurcation as the retrograde velocities were larger. For instance, the ratio of max negative velocity to the maximum positive velocity in Fig 7b and 7d is 1.24% and 19.57%, respectively. Physic informed neural network (PINN) could be an alternate method for correcting this overestimation by predicting the near-wall blood flow [83].

It is worth emphasizing that the velocity values were estimated using the velocities of the particles passing through the POI at different time points. Therefore, larger errors were observed at the times when fewer data points were available at the POI (Fig 7e). Kriging calculates the local uncertainties which will reflect the uncertainties in the estimation of the velocity in locations with data scarcity. Using only raw data from the particles could potentially result in significantly high errors. Another option to correct this error and predict negative velocity is to impose no-slip boundary conditions in the kriging step.

We used the results from Fig 4 to estimate the optimal number of particles and number of released batches as 40 and 15, respectively. In total, we released 600 particles. Using kriging to reconstruct the velocity at a plane allowed us to reduce the need for higher numbers of particles. References [35] reported 8730 registered particles (over 50 minutes) to reconstruct the velocity field in a pinched tube with a pulsatile flow.

The estimation of the area-averaged velocities was similar in Fig 5a and 5d. In both cases, the occlusion level is 50% and the velocities are comparable. The velocities are less in the anatomically accurate case than the velocities in the idealized bifurcation because of different inlet areas and having the same flow rates. Moreover, the anatomical model has seven outlets and one outlet before the stenosis in the same branch. Therefore, it should be noted that if the velocity increases in the anatomical coronary artery model, larger negative velocities after the stenosis and, consequently, higher errors and uncertainties are expected.

We performed convergence analyses for the sampling and particle tracking to determine the number of attempts for random sampling points and particles released. The results showed that the number of attempts for particle tracking is 10, which is less than the corresponding value for sampling, i.e., 30. This is because, in sampling, we selected the points in a circle with 80% of the radius of the vessel at the plane of interest, while in the particle tracking, most of the particles accumulate in the regions with a positive velocity at the POI.

It should be noted that pairing the data in different frames for the reconstruction of the trajectories created by multiple particles from experimental data are very challenging [35], especially when the particles are close to each other in regions in the size of the coronary arteries and may introduce error to final velocity reconstruction. Typically, the particle pairing is done through a modified nearest-neighbors algorithm [20]. However, for rapid flows and relatively low sampling rates, more robust pairing algorithms [35,84] are required. Machine learning techniques based algorithms have been developed to track multiple particles without a priori knowledge of the number of particles, such as the PEPT-ML algorithm [12]. Any gap in the data due to particles not paired to any other particle can be considered a contributor to the sparsity of the data for the Gaussian process.

In the real PEPT data, different incidents are registered with uncontrolled Δ*t*_c_; however, different Δ*t*_c_ would not affect our Gaussian process approach because it is performed in space and time simultaneously. In other words, during the data preparation, before the kriging, the corresponding time for each particle would be unique to that particle.

The particle data scarcity around the wall is the main source of error in our work. Nonetheless, one may consider benefiting from cell or particle margination in blood flow. It has been known that particles with different properties have different cross-streamline migration, also known as margination, in the blood flow [85–89]. Stiff particles were observed to move toward the vessel walls due to their hydrodynamic interaction with softer particles, i.e., red blood cells [90,91]. This behavior is common between synthetic particles and naturally stiffer blood elements such as platelets and white blood cells. Computational approaches have been employed to understand the mechanism and dynamics of margination [89,91–99]. In our study, we did not consider any cross-streamline migration for particle tracking. Nevertheless, the margination can be exploited to gather more data around the wall. One possibility is to use particles with different properties as tracer particles. However, it should be noted that margination decreases as the vessel diameter increase [100]; therefore, the effect is weaker in larger vessels. Other hemodynamic and geometric quantities such as wall shear rate, vessel orientation and pulsatility of the flow also affect the margination behavior of microparticles [100].

In our study, with a full in-silico approach, we have particularly focused on the blood velocity reconstruction in the coronary artery with stenosis. We wanted to test the plausibility of recovering blood flow profiles using PEPT, which is a potential novel imaging application of this technology. We have demonstrated how Gaussian processes can be used to recover in the flow from temporal-spatial sparse data simultaneously and have identified potential limitations in measuring the velocity of fast biofluids with potentially negative velocity.

### Limitations

We presented a simulation workbench to evaluate a novel imaging modality. Simulations provide an approximation of the system under study and have inherent limitations. First of all, one should note that there are other factors, such as the interaction between the particles, resolution of the gamma-ray detector and particle size, that could affect the accuracy of the PEPT measurement, stenosis diagnosis, and velocity profile reconstruction. In this paper, we focused on particle delivery in PEPT. Another limitation is that our analysis for the anatomically accurate model was done for one case. The coronary tree has large inter-individual variation. Therefore, to quantify the uncertainty of PEPT measurement, a larger cohort of patient-specific anatomies is needed. Due to the lack of pressure and flow measurement for the patient, we could not use patient-specific boundary conditions. Nevertheless, we used an optimized lumped parameter model for which the pressures and flow rates were within the physiological range. Moreover, we assumed that the locations of the particles are measured instantaneously and accurately, ignoring the false detection of the gamma rays that may happen in practice. The boundary of the domain was also assumed segmented from CT data in the patient-specific case, which would be possible only if PEPT data were coupled with MRI or CT data. Displacement and deformation of the computational domain were not measured directly from the CT images and were neglected to simplify the complexity and assess the accuracy of the proposed Gaussian process approached by comparing the reconstructed velocity to the CFD results. The displacement and deformation of the coronary artery will introduce additional sources of error [101] that may require further algorithm developments. In this study, we have performed a computational experiment on PEPT accuracy, flow recovery and uncertainty. The next step is to quantify errors due to the tracer activity, positron range, false detection, gamma ray scattering, particle size effect, etc. This can be achieved using Geant4 (GATE), however, these results will depend on the scanner and particle size, which will require further characterization. Our expectation for PEPT is that we rely on small particles or labelled cells, which will have a nominal impact on the flow. It is important to note that as particles get larger, they may impact blood flow and this could introduce new sources of error, which will decrease the accuracy of the recovered flow field (see Fig 8). As PEPT measurement in biology is a novel and under-development medical measurement technique, the significance of various types of error is still unknown. However, future work will consider different sources of errors in PEPT measurement to quantify their effect.

## Conclusion

This study is a proof-of-concept study for using PEPT technology to diagnose coronary occlusion. We used CFD techniques to generate the blood flow in a straight tube and a bifurcation with occlusion and then reconstructed the velocity profile after the occlusion using the kriging process. For reconstructing the velocity profile, we used the data from sampling and particle tracking in the flow field. We could reconstruct the velocity profile using a limited number of particles released upstream of the location of interest. The reconstructed profiles can be used for the calculation of the wall shear stress and FFR if needed. We conclude that kriging can be used to estimate the maximum cross-sectional velocity after the stenosis but fails to reconstruct the velocity profiles accurate; especially in cases with large near-wall negative velocity. This shows that PEPT can feasibly be developed to provide a novel measurement of blood flow profiles for imaging and cardiac diagnosis.

## Supporting information

S1 Text Universal kriging. (DOCX)

S1 Table The lump parameters constants used for the boundary conditions for idealised bifurcation. The units for the resistance values and the compliance values are [mmHg·s/cm3] and [cm3/mmHg], respectively.
(DOCX)

S2 Table The lump parameters constants used for the boundary conditions for anatomically-accurate geometry. The units for the resistance values and the compliance values are [mmHg·s/cm3] and [cm3/mmHg], respectively.
(DOCX)
