# Supplementary material for: Using Gaussian process for velocity reconstruction after coronary stenosis applicable in positron emission particle tracking: An in-silico study
Source: PLoS One. 2023 Dec 14;18(12):e0295789. doi: 10.1371/journal.pone.0295789 (PMC10721050; doi:10.1371/journal.pone.0295789)
Supplement: S1 Text — (DOCX) [file pone.0295789.s001.docx]

**S1 Text**

**Universal kriging**

The velocity $v$ at the location $\mathbf{x}$ can be decomposed as:

| $v(\mathbf{x})=\mu(\mathbf{x})+\varepsilon(\mathbf{x})$, | (A) |
| --- | --- |

where $\mu$ and $\varepsilon$ are the unknown mean and the unknown variation.

The value at a location where no data is measured, $\hat{\mathbf{x}}$, can be predicted as a weighted interpolation between all data as

| $v\left( \hat{\mathbf{x}} \right)=\sum_{i}^{N} \left( \omega_{i}v\left( \mathbf{x}_{i} \right) \right),$ | (B) |
| --- | --- |

where $\omega_{i}$ is the optimal weight corresponding to the data gathered at $\mathbf{x}_{i}$. The weights are calculated in a minimization problem using the Lagrange multiplier method [1]:

| $\left\{ \begin{aligned} \gamma\left( x_{i},\hat{x} \right) =\sum_{j=1}^{N} \omega_{j}\gamma\left( \mathbf{x}_{i},\mathbf{x}_{j} \right)+\sum_{l=1}^{k} \lambda_{l}f_{l}\left( \mathbf{x}_{i} \right) i=1,2,\ldots,N \\ f_{l}\left( \hat{x} \right) =\sum_{i=1}^{N} \omega_{i}f_{l}\left( \mathbf{x}_{i} \right) l=1,2,\ldots,k \end{aligned} \right.$ | (C) |
| --- | --- |

where $\lambda$ is the Lagrange multiplier, $\gamma$ is the covariance and $f\left( \mathbf{x}_{i} \right)$ is the measured values at $\mathbf{x}_{i}$.

The local variance at grid points, $\sigma_{k}^{2}(\hat{\mathbf{x}})$, is then calculated as

| $\sigma_{k}^{2}\left( \hat{\mathbf{x}} \right)=\sum_{i=1}^{N} \omega_{i}\gamma\left( \mathbf{x}_{i},\hat{\mathbf{x}} \right)+\sum_{l=1}^{k} \lambda_{l}f_{l}\left( \hat{\mathbf{x}} \right).$ | (D) |
| --- | --- |

Boundary conditions, i.e., zero velocity, were then imposed at the boundary of planes of interest before performing kriging to compute the velocities at locations where no sampled data was available.

**Reference**

1. Kumar V. Optimal contour mapping of groundwater levels using universal kriging - A case study. Hydrol Sci J. 2007;52: 1038–1050. doi:10.1623/hysj.52.5.1038
