# Supplementary material for: Using Gaussian process for velocity reconstruction after coronary stenosis applicable in positron emission particle tracking: An in-silico study
Source: PLoS One. 2023 Dec 14;18(12):e0295789. doi: 10.1371/journal.pone.0295789 (PMC10721050; doi:10.1371/journal.pone.0295789)
Supplement: S1 Table — The units for the resistance values and the compliance values are [mmHg·s/cm3] and [cm3/mmHg], respectively. (DOCX) [file pone.0295789.s002.docx]

**S1 Table.** The lump parameters constants used for the boundary conditions for idealised bifurcation. The units for the resistance values and the compliance values are [mmHg·s/cm^3^] and [cm^3^/mmHg], respectively.

|  | $R_{v}$ | $R_{a}$ | $R_{a-m}$ | $C_{a}$ | $C_{\mathrm{im}}$ |
| --- | --- | --- | --- | --- | --- |
| **LAD** | 9.695 | 5.658 | 4.687×10 | 3.952×10^-3^ | 3.807×10^-2^ |
| **LCx** | 1.127×10 | 5.502×10^-1^ | 6.005×10 | 3.379×10^-3^ | 4.483×10^-2^ |
