# Supplementary material for: Using Gaussian process for velocity reconstruction after coronary stenosis applicable in positron emission particle tracking: An in-silico study
Source: PLoS One. 2023 Dec 14;18(12):e0295789. doi: 10.1371/journal.pone.0295789 (PMC10721050; doi:10.1371/journal.pone.0295789)
Supplement: S2 Table — The units for the resistance values and the compliance values are [mmHg·s/cm3] and [cm3/mmHg], respectively. (DOCX) [file pone.0295789.s003.docx]

**S2 Table.** The lump parameters constants used for the boundary conditions for anatomically-accurate geometry. The units for the resistance values and the compliance values are [mmHg·s/cm^3^] and [cm^3^/mmHg], respectively.

|  | $R_{v}$ | $R_{a}$ | $R_{a-m}$ | $C_{a}$ | $C_{\mathrm{im}}$ |
| --- | --- | --- | --- | --- | --- |
| **LC1** | 1.243×10^2^ | 3.371×10^2^ | 5.900×10^2^ | 7.558×10^-4^ | 4.220×10^-3^ |
| **LC2** | 2.324×10 | 2.439×10 | 1.104×10^2^ | 8.581×10^-4^ | 2.269×10^-2^ |
| **LAD1** | 3.107×10^2^ | 7.009×10^2^ | 9.990×10^2^ | 1.574×10^-4^ | 2.966×10^-3^ |
| **LAD2** | 1.272×10^2^ | 3.593×10^2^ | 6.476×10^2^ | 1.688×10^-4^ | 5.006×10^-3^ |
| **LAD3** | 1.408×10^2^ | 4.340×10 | 7.091×10^2^ | 3.568×10^-4^ | 2.532×10^-3^ |
| **LAD4** | 4.716×10 | 1.092×10 | 2.153×10^2^ | 1.002×10^-3^ | 4.080×10^-3^ |
| **LAD5** | 5.589×10 | 1.367×10^2^ | 2.701×10^2^ | 1.032×10^-4^ | 6.745×10^-3^ |
